# Frontotemporal dementia with right temporal predominance: a clinical comparison with left-predominant FTD

**DOI:** 10.1007/s00415-026-14042-2

**Published:** 2026-08-07

**Authors:** Jennifer C. Thompson, Christopher Kobylecki, Matthew Jones, Jessica Haigh, Matthew Larbey, Tobias C. Langheinrich, Rachel Thomasson, Anna M. T. Richardson, Julie S. Snowden

**Affiliations:** 1 Cerebral Function Unit Manchester Centre for Clinical Neurosciences, Salford Royal Hospital, Manchester Academic Health Sciences Centre, Northern Care Alliance NHS Foundation Trust, Salford, M6 8HD UK; 2 Division of Psychology, Communication and Human Neuroscience, Faculty of Biology, Medicine and Health, University of Manchester, Manchester, UK; 3 Division of Neuroscience, Faculty of Biology, Medicine and Health, University of Manchester, Manchester, UK; 4 Division of Medical Education, Faculty of Biology, Medicine and Health, University of Manchester, Manchester, UK

**Keywords:** Frontotemporal dementia, Semantic dementia, Primary progressive aphasia, Semantic variant PPA, Right-temporal variant frontotemporal dementia

## Abstract

**Background:**

The right-temporal variant of frontotemporal dementia (FTD) is well characterised. Whether it should be considered a distinct clinical entity, separate from other syndromes of FTD, remains an open question. The study addressed the issue through a retrospective comparison of clinical characteristics of patients with predominant atrophy in right or left anterior temporal lobe (R-ATL vs. L-ATL).

**Methods:**

Patients were identified from a clinical database, diagnosed with FTD, and reported to show temporal lobe atrophy on imaging. Fifty-one patients were selected in whom independent ratings of atrophy were greatest in right or left anterior temporal lobe. Presenting symptoms, cognitive and behavioural characteristics, neuropsychological findings, and diagnostic classification were recorded.

**Results:**

Difficulty recognising people, impaired decision-making, perseverative preoccupations, disinhibition, and loss of empathy characterised the R-ATL group, in keeping with the previous reports. There was, however, overlap in cognitive and behavioural symptomatology in R-ATL and L-ATL, and sensitivity and specificity values were modest. Group differences diminished with disease progression. The most common clinical classification at first assessment in both groups was semantic dementia (SD), with other patients being classified as behavioural-variant FTD (bvFTD), FTD with amyotrophic lateral sclerosis or mixed FTD/SD. Not all patients with L-ATL met criteria for semantic variant primary progressive aphasia (svPPA).

**Conclusions:**

The data question the notion that R-ATL and L-ATL presentations are separate entities. We argue that a common diagnostic framework for the two is warranted, with classification being based on cognitive/behavioural characteristics rather than neuroradiological grounds.

## Introduction

Recent years have seen increased attention on a form of frontotemporal dementia (FTD) associated with atrophy predominantly affecting the right-anterior temporal lobe (R-ATL). Impaired recognition of people, compulsions/preoccupations, and loss of empathy have been identified as core characteristics [1–3], and a multi-centre international cohort study of 360 patients [4] demonstrated, in addition, high frequencies of apathy, behavioural disinhibition, naming difficulty, and memory impairment.

Studies of FTD with R-ATL predominance are immensely valuable. First, they draw attention to a presentation of FTD that may be under-recognised. Second, they potentially improve understanding of the role of the right temporal lobe in cognition and behaviour. They also pose a fundamental question of nosology. How should diverse clinical presentations be accounted for within clinical diagnostic frameworks for FTD? It has been argued that R-ATL predominant FTD should be considered a distinct clinical entity, separate from its left-temporal lobe (L-ATL) counterpart, semantic variant primary progressive aphasia (svPPA). The designation “semantic behavioural variant FTD” has been proposed to capture the mixed picture of behavioural and semantic symptoms [2]. The term ‘semantic dementia’ (SD) has, however, long been used to encompass patients with both left and right-predominant ATL atrophy [5–7], and behavioural change has been acknowledged as an integral component of the syndrome [8, 9]. An implicit assumption in using this term is that left- and right-predominant ATL are variants of the same underlying clinicopathological entity.

An important consideration in addressing the relationship between R and L-ATL is the extent to which features that characterise R-ATL FTD are shared by patients with L-ATL predominance. Patients with R-ATL predominance are, thus far, identified purely on radiological grounds. It is essential that comparisons should involve L-ATL predominant patients also selected on radiological grounds rather than a specific FTD subvariant, e.g., svPPA. The present study aimed to provide such a comparison. It comprised a retrospective examination of information recorded in case notes of patients attending a specialist diagnostic clinic for early onset and rare dementias. It was enabled by the fact that clinic assessments involve a structured clinical history of cognitive and behavioural changes and neuropsychological assessment, so that comparable information is available for all patients, recorded independently and without prior knowledge of neuroradiological findings.

## Methods

### Identification of study group

Patients were identified from our clinical research database, who were assessed between 2006 and 2023, clinically diagnosed with any form of FTD and reported to have predominant temporal lobe atrophy on structural magnetic resonance (MR) imaging. They were included only if clinical, behavioural, and neuropsychological data were available and suitable coronal images of MR scans were accessible for rating. The coronal images were then examined by an independent neuroradiological expert, blind to patients’ clinical characteristics. Scans were rated for severity of atrophy in frontal and anterior temporal lobes using the 5-point visual rating scale of Kipps et al. [10], which ranges from 0 (no atrophy) to 4 (severe atrophy). Patients were selected who had disproportionate atrophy in either right or left ATL, defined by a severity rating greater than in the contralateral anterior temporal lobe, and both frontal lobes.

### Review of clinical case notes

The following information was recorded from patients’ clinical files, relating to their initial diagnostic work-up:

- Presenting complaints. Patients and/or their caregiver/informant had been asked to state their principal concerns in order of importance, with up to three presenting complaints recorded for each patient. These self-generated symptoms were mapped onto the following domains: personality and behaviour, naming, word comprehension, people recognition/naming, and memory.
- Cognitive symptoms, based on clinical interview. The interview addressed problems in naming and word comprehension, recognition of people, recognition of objects, topographical orientation, calculation, visuospatial skills, memory, and planning, judgement, and decision-making.
- Behavioural, affective, and neuropsychiatric changes, based on clinical interview and clinical observations. Features recorded included affective blunting, exuberant/over-demonstrative affect, childish manner, disinhibited behaviour, reduced empathy, simple repetitive behaviours, complex repetitive behaviours, routine and/or time-bound behaviour, perseverative preoccupations, hypersensitivity to sensory stimuli, somatisation, sweet-food preference, restrictive food preference, reduced self-care, altered self-care, social avoidance, low mood, and anxiety.
- Neuropsychological test findings. Patients underwent neuropsychological assessment as part of their diagnostic work-up. Results were recorded for naming (Graded Naming Test, Manchester Naming Test), word comprehension (Manchester word-picture matching test), digit span (forwards and reverse), verbal fluency (semantic fluency: number of animals produced in 60 s; letter fluency: total number of words beginning with F, A and S, respectively in 60 s), famous face identification and naming (using 10 high profile famous faces), perceptuospatial function (incomplete letters, dot counting, and cube analysis subtests from the Visual Object and Space Perception Battery), memory (using the Visual Object Memory Test [11] which comprises immediate and delayed free recall and 4-choice visual recognition of pictured objects), and executive function (Weigl Colour-Form Sorting Test).

Clinical features emerging on follow-up of patients were recorded. The clinical diagnostic classification of each patient, based on cognitive, behavioural, and neurological features following initial assessment, was documented. Whether patients fulfilled current diagnostic criteria for bvFTD [12] and svPPA [13] was also recorded. Genetic and pathological data were sparse but are reported where available.

### Statistical analysis

Data were analysed using IBM SPSS Statistics Version 29. Demographic characteristics were compared using t tests or χ^2^ tests. Categorical data were analysed using χ^2^, with Cramér’s V (Φ) reported for effect sizes. Neuropsychological test findings were compared using non-parametric Mann–Whitney *U* tests, with *r* (*z/* √ *n*) reported for effect sizes. Planned group comparisons were regarded as noteworthy where effect size was > 0.3 or *p* value < 0.05.

### Ethics statement

Data used in the present study were collected as part of patients’ routine clinical care. Patients, or their consultees, provided written informed consent, in accordance with the Declaration of Helsinki, for their clinical information to be included in a clinical research database and to be used for research purposes. The clinical research database was approved by the Newcastle and Tyne Research Ethics Committee (REC reference 19/NE/0219, IRAS project ID 266294, project title “Clinical data in research into neurodegenerative disorders”).

## Results

### Patients and demographics

A total of 51 patients were identified with disproportionate atrophy in either the right or left anterior temporal lobe: 25 with right-predominant ATL atrophy (R-ATL) and 26 with left-predominant ATL atrophy (L-ATL). Further 5 patients had equal right- and left-rated ATL predominant atrophy so were excluded from further analysis. The R-ATL group were older at the time of symptom onset and initial evaluation but neither difference reached statistical significance. No differences between R-ATL and L-ATL groups were observed in duration of illness at presentation, handedness, or presence of a family history of neurodegenerative disease (Table 1). 73% of the cohort were followed longitudinally (76% R-ATL, 69% L-ATL), the median period of follow-up being 3 years in the R-ATL group and 4 years in the L-ATL group.

**Table 1:** Demographic characteristics of R-ATL vs L-ATL patients

	R-ATL	L-ATL	*t* test or χ^2^	*P* value
Age	66.4 (7.1)	61.9 (9.9)	*t* = 1.9	0.06
Age at onset	63.6 (7.6)	58.6 (9.6)	*t*= 2.0	0.05
Duration at presentation	3.2 (1.6)	2.8 (1.5)	*t* = −0.7	0.51
Sex (M/F)	14/11	16/10	χ^2^ = 0.16	0.68
Handedness (R/L)	23/2	25/1	χ^2^ = 0.40	0.53
Family history	40%	23%	χ^2^ = 1.70	0.19
Autosomal dominant	8%	0%	χ^2^ = 2.17	0.14

*Family history* any family history of neurodegenerative dementia, *Autosomal dominant * at least 3 affected individuals over two generations with at least one being a first degree relative of the other two

### Visual ratings of ATL atrophy

Visually rated atrophy scores for right and left anterior temporal lobe atrophy are shown in Table 1. Most patients had atrophy in both right and left ATL, with just one L-ATL patient having an atrophy rating of 0 in the right ATL. Right-left ATL atrophy ratings differed by one scale point in 80% patients and by two scale points in 20% of patients (Table 2).

**Table 2:**
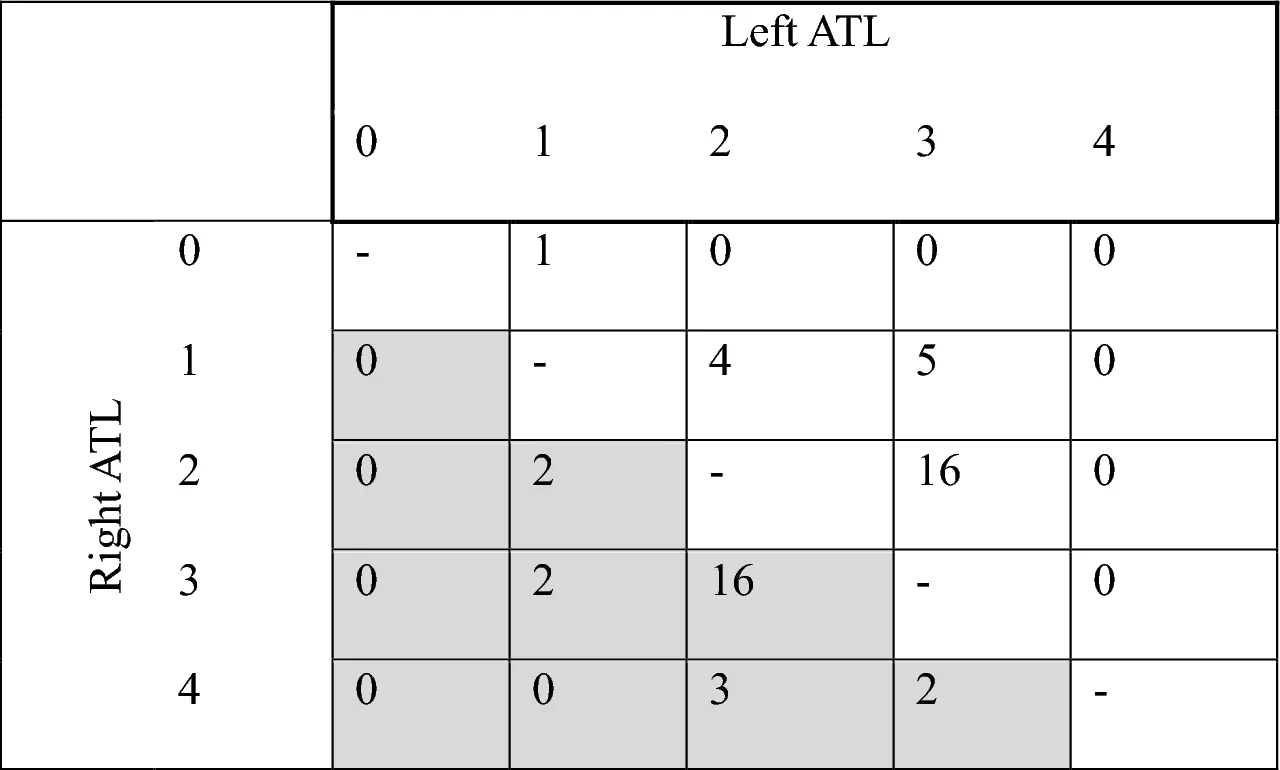
Cross-tabulated frequencies of Kipps–Davies visual ratings for right and left ATL atrophy

ATL atrophy scores 0 (no atrophy) to 4 (severe atrophy). Shaded cells = R-ATL predominant, non-shaded cells = L-ATL predominant,— = non-viable rating

### Presenting complaint in R-ATL and L-ATL

Personality and behavioural change as a presenting complaint was more likely to be associated with R-ATL, whereas impaired naming was associated with L-ATL atrophy (Table 3). There was no statistical difference between groups in patient or caregiver report of poor word comprehension, recognising/naming people, or memory.

**Table 3:** Presenting complaints in R-ATL vs L-ATL

	R-ATL	L-ATL	χ^2^	*P* value	Φ (95% CI)
Personality/behaviour	84	42	9.48	**0.002**	**– 0.43 (– 0.63 to – 0.18)**
Naming	36	73	7.08	**0.008**	**0.37 (0.11–0.59)**
Word comprehension	12	4	1.00	0.317	0.14 (**– **0.14 to 0.40)
People naming/recognition	24	8	2.56	0.109	**– **0.22 (**– **0.47 to 0.05)
Memory	28	8	3.62	0.057	**– **0.27 (**– **0.50 to 0.01)

Frequencies represent percentage of people in whom symptom reported. Effect sizes > 0.3 and significant *p *values are highlighted in bold

### Cognitive and behavioural features elicited on structured interview

The percentage of patients in whom each cognitive and behavioural abnormality was reported at baseline evaluation and over the course of follow-up is shown in Table 4. At initial presentation, difficulty recognising people, exuberant over-demonstrative affect, and perseverative preoccupations were more commonly reported in the R-ATL group, whereas word-finding difficulty and impaired single word comprehension were more associated with L-ATL predominant atrophy. Disinhibited behaviour, reduced empathy, routine-bound behaviour such as clockwatching, and impaired planning, judgement, and decision-making were common in both groups. When perseverative preoccupations and clockwatching behaviours are combined, this was present in 72% R-ATL & 58% L-ATL; the group difference is no longer significant (χ^2=^ = 1.14, *p* = 0.29, Φ = 0.150).

**Table 4:** Symptoms reported on clinical interview at presentation and follow-up in R-ATL and L-ATL

	Presentation					Follow-up				
	R-ATL	L-ATL	Χ^2^	*P* value	Φ (95% CI)	R-ATL	L-ATL	Χ^2^	*P* value	Φ (95% CI)
*Cognition*										
Memory	28	16	1.05	NS	**– **0.15 (**– **0.40 to 0.14)	40	16	1.65	NS	**– **0.21 (**– **0.46 to 0.07)
Word-finding	36	85	12.6	***P* < 0.001**	**0.50 (0.26–0.68)**	52	85	4.86	***P* = 0.012**	**0.36 (0.10–0.58)**
Word comprehension	28	56	4.02	***P* = 0.045**	**0.28 (0.01–0.52)**	56	76	5.7	NS	0.40 (0.14–0.61)
Recognising people	68	39	4.46	***P* = 0.035**	**– 0.30 (– 0.53 to – 0.02)**	76	65	0.11	NS	0.02 (**– **0.26 to 0.29)
Recognising objects	16	20	0.14	NS	0.05 (**– **0.23 to 0.32)	56	52	0.131	NS	0.06 (**– **0.22 to 0.33)
Topographical orientation	8	4	0.36	NS	**– **0.08 (**– **0.35 to 0.20)	8	8	0.18	NS	0.02 (**– **0.25 to 0.30)
Planning, judgement, decision-making	71	46	3.12	NS	**– **0.25 (**– **0.49 to 0.03)	83	58	3.70	***P* = 0.048**	**0.33 (0.06–0.56)**
Calculation	4	4	0.003	NS	**– **0.01 (**– **0.28 to 0.27)	8	4	1.80	NS	**– **0.22 (**– **0.47 to 0.06)
Visuospatial skills	4	0	1.06	NS	**– **0.14 (**– **0.40 to 0.14)	4	0	0.87	NS	**– **0.15 (−0.41 to 0.13)
*Affect and behaviour*										
Blunting	24	12	1.36	NS	**– **0.16 (**– **0.42 to 0.12)	28	19	0.03	NS	**– **0.03 (**– **0.30 to 0.25)
Exuberant, over-demonstrative	32	8	4.77	***P* = 0.029**	**– 0.31 (– 0.54 to – 0.03)**	36	12	2.20	***P* = 0.040**	**– 0.24 (– 0.49 to 0.03)**
Childish, fatuous	16	4	2.13	NS	**– **0.20 (**– **0.45 to 0.08)	16	12	0.47	NS	0.04 (**– **0.24 to 0.31)
Disinhibited	56	46	0.49	NS	**– **0.10 (**– **0.36 to 0.18)	64	65	0.74	NS	**– **0.17 (**– **0.42 to 0.11)
Reduced empathy	58	48	0.53	NS	**– **0.10 (**– **0.37 to 0.18)	63	60	0.03	NS	**– **0.03 (**– **0.30 to 0.25)
Simple repetitive behaviour	16	8	0.85	NS	**– **0.13 (**– **0.39 to 0.15)	20	15	0.03	NS	0.04 (**– **0.24 to 0.31)
Complex repetitive behaviour	32	15	1.96	NS	**– **0.20 (**– **0.45 to 0.08)	36	31	0.62	NS	0.16 (**– **0.12 to 0.42)
Routine and/or time-bound behaviour	32	46	1.07	NS	0.15 (**– **0.14 to 0.40)	64	73	1.21	NS	0.18 (**– **0.10 to 0.44)
Perseverative preoccupations	72	32	8.01	***P* = 0.005**	**−0.40 (– 0.61 to – 0.14)**	88	56	6.70	**P = 0.012**	**– 0.43 (– 0.63 to – 0.18)**
Hypersensitivity to sensory stimuli	16	12	0.17	NS	**– **0.06 (**– **0.33 to 0.22)	44	24	0.54	NS	**– **0.12 (**– **0.38 to 0.16)
Somatisation	24	12	1.22	NS	**– **0.16 (**– **0.41 to 0.12)	40	28	0.09	NS	**– **0.05 (**– **0.32 to 0.23)
Sweet food preference	32	36	0.89	NS	0.04 (**– **0.24 to 0.31)	64	52	0.55	NS	**– **0.12 (**– **0.38 to 0.16)
Restrictive food preference	36	32	0.89	NS	**– **0.04 (**– **0.31 to 0.24)	56	64	0.29	NS	0.09 (**– **0.19 to 0.36)
Reduced self-care	36	32	0.89	NS	**– **0.04 (**– **0.31 to 0.24)	56	44	0.62	NS	**– **0.13 (**– **0.39 to 0.15)
Altered self-care	16	0	4.35	NS	**– **0.30 (**– **0.53 to **– **0.02)	20	8	0.89	NS	**– **0.16 (**– **0.41 to 0.12)
Social avoidance	21	12	0.70	NS	**– **0.12 (**– **0.38 to 0.16)	21	20	0.18	NS	**– **0.07 (**– **0.34 to 0.21)
Low mood	16	8	0.85	NS	**– **0.13 (**– **0.39 to 0.15)	20	8	0.87	NS	**– **0.15 (**– **0.41 to 0.13)
Anxiety	16	4	2.13	NS	**– **0.20 (**– **0.45 to 0.08)	20	8	0.82	NS	**– **0.05 (**– **0.32 to 0.23)

Frequencies represent percentage of people who endorsed symptom. Effect sizes > 0.3 and significant *p *values are highlighted in bold

Differences between the R-ATL and L-ATL groups diminished over time. Difficulty recognising people was no longer significantly different. Behavioural differences were also less marked; however, impaired planning, judgement, and decision-making became more common in R-ATL over time.

### Neuropsychological assessment in R-ATL and L-ATL

The L-ATL group performed more poorly on tests of naming, including for low-frequency (Graded Naming Test) and high-frequency (Manchester Naming Test) objects, and famous faces, as well as single word comprehension and verbal fluency (category and letter), as shown in Table 5. However, both groups demonstrated high frequencies of impairment relative to normative data in naming and face identification.

**Table 5:** Neuropsychological task performance in R-ATL vs L-ATL

	R-ATL			L-ATL			Significance		
	*n*	Median (range)	% impaired	*n*	Median (range)	% impaired	Z	*P* value	*r* (95% CI)
MMSE	25	24 (10–29)	44	25	24 (6–30)	44	– 0.661	0.508	– 0.09 (– 0.36 to 0.19)
Graded Naming Test/30	24	2 (0–15)	92	25	0 (0–21)	95	– 3.2	**0.001**	– **0.46 (**– **0.65 to −0.20)**
Manchester Naming Test/40	25	29 (11–39)	72	26	14.5 (0–40)	92	– 3.697	** < 0.001**	– **0.52 (**– **0.69 to **– **0.28)**
Word-Picture Matching/40	21	40 (32–40)	43	23	36 (0–40)	74	– 2.781	**0.005**	– **0.42 (**– **0.64 to −0.14)**
Digit span forwards	25	7 (4–9)	4	25	6 (4–8)	8	– 1.498	0.134	– 0.21 (– 0.46 to 0.07)
Digit span reverse	25	4 (2–7)	12	24	4 (0–7)	8	– 0.051	0.959	– 0.01 (– 0.29 to 0.27)
Category fluency	24	9 (3–18)	63	26	5 (0–17)	85	– 2.901	**0.004**	– **0.41 (**– **0.62 to **– **0.15)**
Letter fluency	24	21 (5–40)	33	26	13 (0–32)	77	– 2.226	**0.026**	– **0.31 (**– **0.55 to **– **0.04)**
Famous faces identified/10	24	6 (2–10)	58	25	6 (0–10)	56	– 0.999	0.318	– 0.14 (– 0.41 to 0.14)
Famous faces named/10	24	4.5 (0–10)	63	25	2 (0–10)	92	– 3.265	**0.001**	– **0.47 (**– **0.66 to **– **0.21)**
VOSP incomplete letters/20	24	19 (2–20)	25	25	19 (0–20)	24	– 0.042	0.967	– 0.01 (– 0.29 to 0.28)
VOSP silhouettes/30	12	9 (2–14)	100	10	7 (0–20)	80	– 0.894	0.371	– 0.19 (– 0.57 to 0.25)
VOSP dot counting/10	24	10 (7–10)	4	25	10 (10–10)	0	– 2.38	**0.017**	– **0.34 (**– **0.57 to **– **0.07)**
VOSP cube analysis/10	23	9 (3–10)	4	25	10 (7–10)	0	– 1.452	0.146	– 0.21 (– 0.47 to 0.08)
VoM immediate recall/20	24	3 (0–10)	92	25	1 (0–10)	96	– 1.503	0.133	– 0.21 (– 0.47 to 0.07)
VoM immediate recognition/20	24	16.5 (5–20)	50	24	19 (6–20)	25	– 2.038	**0.042**	– **0.29 (**– **0.53 to **– **0.01)**
VoM delayed recall/20	23	2 (0–10)	74	25	0 (0–20)	76	– 0.991	0.322	– 0.14 (– 0.41 to 0.15)
VoM delayed recognition/20	23	14 (6–20)	61	24	18.5 (5–20)	21	– 2.67	**0.008**	– **0.39 (**– **0.61 to **– **0.12)**
Weigl block sort/9	23	9 (2–9)	39	26	8 (3–9)	39	– 0.524	0.6	– 0.07 (– 0.35 to 0.21)

*VOSP*Visual Object and Space Perception, *VoM* Visual Object Memory, effect sizes > 0.3 and significant *p *values are highlighted in bold

The R-ATL group performed less well on the Visual Object Memory immediate and delayed 4-choice visual recognition tasks. R-ATL patients showed poorer performance on spatial tasks, although the significance is tempered by the observation that only 4% were below the cut-off for impairment for dot counting and cube analysis.

### Sensitivity and specificity of cognitive and behavioural features reported to be characteristic of right-ATL atrophy

**Fig. 1:**
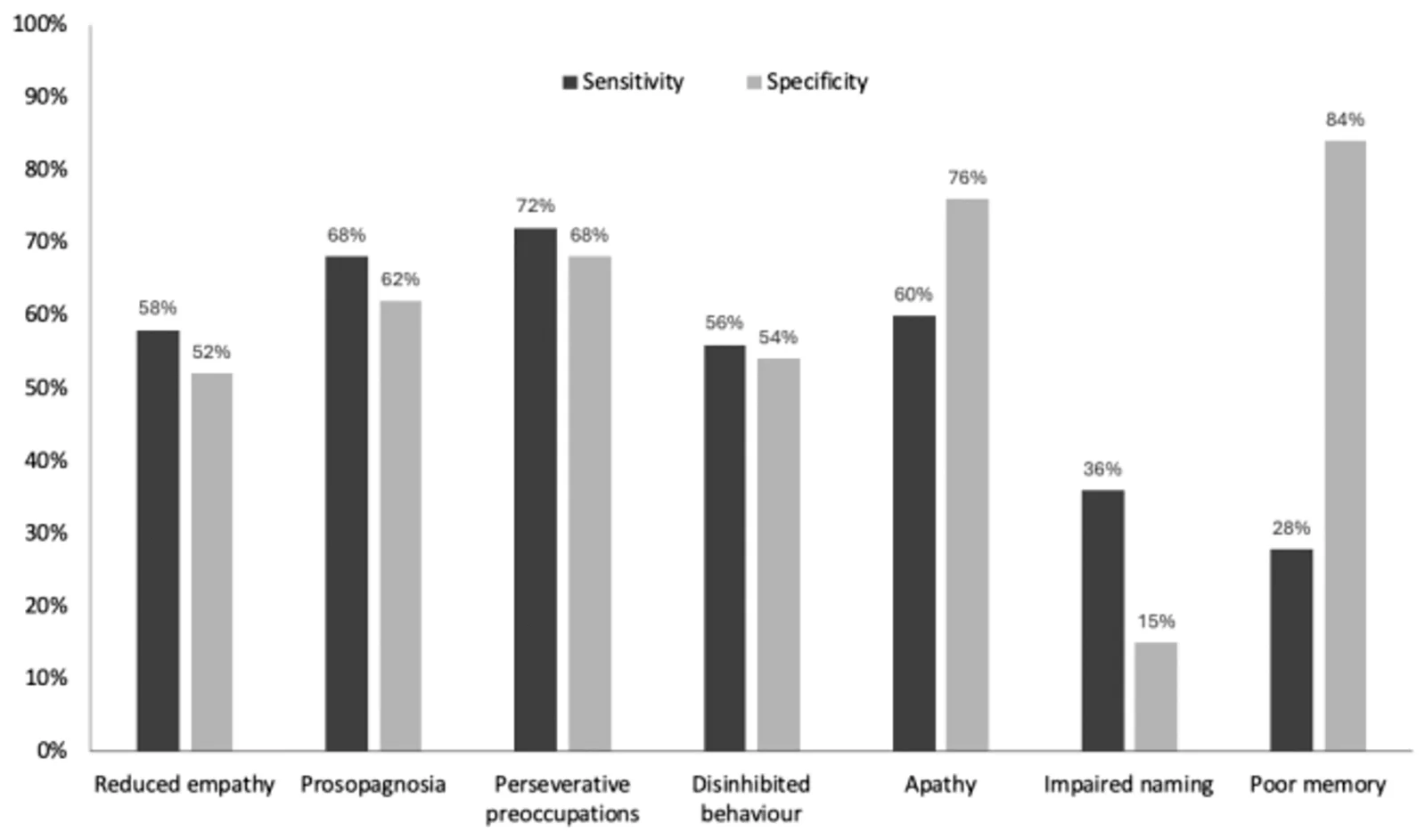
Sensitivity and specificity of clinical features reported to be characteristic of R-ATL

Sensitivity and specificity were calculated for clinical features previously reported to be characteristic of right-temporal-variant FTD [4], within the cohort of right-or-left-predominant ATL FTD, and are shown in Fig. 1. Perseverative preoccupation was the feature with highest sensitivity. Poor memory showed low sensitivity, but high specificity. Overall rates of sensitivity were low, with no item above 80%.

### Clinical diagnostic classification

**Fig. 2:**
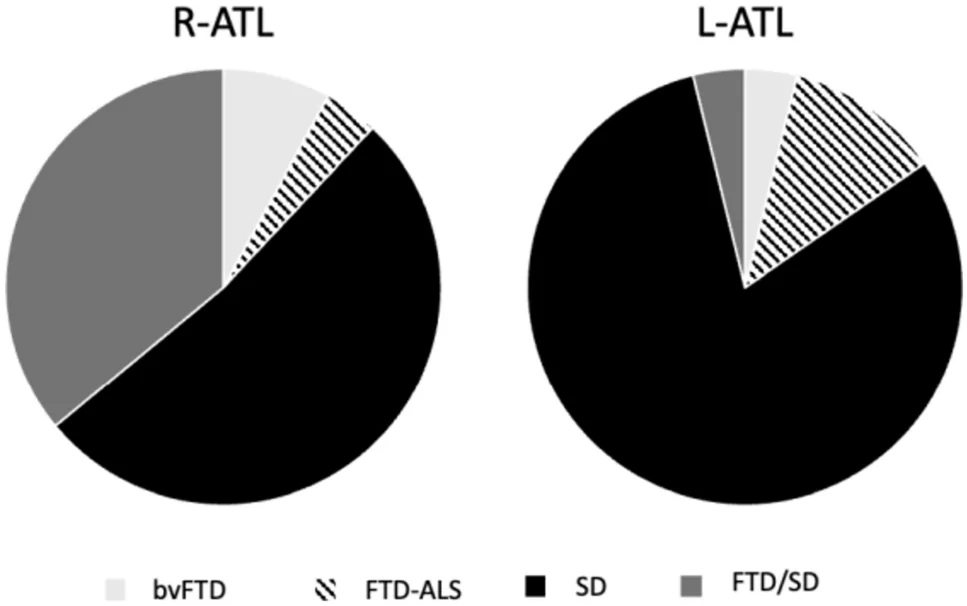
Clinical diagnostic classification in R-ATL vs L-ATL

SD was the most common designation in both R-ATL and L-ATL groups (Fig. 2). Nevertheless, there was phenotypic variation in both groups. Two R-ATL and one L-ATL patients were classified as bvFTD. One R-ATL and three L-ATL patients were classified as FTD-ALS, based on the presence of FTD with amyotrophic lateral sclerosis (ALS). Nine R-ATL and one L-ATL patient showed semantic impairment, in the context of prominent behavioural disorder, designated FTD/SD.

### Application of current diagnostic criteria for bvFTD and svPPA

96% (24/25) R-ATL patients had at least one bvFTD behavioural criteria feature, compared with 69% (18/26) of L-ATL patients (χ^2=^ = 7.6, *p* = 0.006, Φ = – 0.385, 95% CI – 0.60 to – 0.12). R-ATL patients were more likely to meet diagnostic criteria for bvFTD (64%) than L-ATL patients (35%) (χ^2=^ = 4.4, *p* = 0.036, Φ = – 0.294, 95% CI – 0.29 to – 0.02). 84% (21/26) of L-ATL patients met diagnostic criteria for svPPA compared with 56% (14/25) of R-ATL cases (χ^2=^ = 3.6, *p* = 0.057, Φ = – 0.267, 95% CI – 0.01 to 0.51). However, some patients who met criteria for svPPA did not meet all-cause PPA criteria [14], as language impairment was not the principal clinical feature or basis for impaired ADLs. Just 54% (14/26) of L-ATL cases and 20% (5/25) of R-ATL cases met criteria for all-cause PPA and svPPA (χ^2=^ = 6.2, *p* = 0.020, Φ = – 0.350, 95% CI 0.08 to 0.57).

### Genetics and pathology

Genetic and pathological data were sparse. Clinical genetic tests were not routinely carried out in the absence of a positive family history, hence in most SD patients. However, one R-ATL patient with positive family history and clinical designation of FTD/SD had an *MAPT* mutation. Another R-ATL patient with bvFTD had a *GRN* mutation and associated TDP-A pathology. A third R-ATL patient with clinical SD had TDP-type C pathology.

## Discussion

The most frequently reported symptoms in R-ATL patients were difficulty recognising people, impaired planning and decision-making, perseverative preoccupations, disinhibition, and loss of empathy. These findings mirror closely those of independent studies [1, 2, 15, 16] including a multi-centre international collaboration [4]. The prominence of person recognition impairment in the R-ATL group, contrasting with naming and word comprehension problems in the L-ATL group, accords with the large body of evidence that the right- and left-temporal lobes contribute differentially to semantic memory [6, 7, 17–22]. The greater difficulty in visual object recognition memory in the R-ATL group might suggest impaired object semantics associated with R-ATL degeneration. The prominence of behavioural symptoms associated with R-ATL atrophy is well documented [15, 23–25] and in keeping with evidence that the right temporal lobe has a prominent role in social function and theory of mind [26].

Notwithstanding those findings, this study also underscores the fact that left–right distinctions are relative rather than absolute. Problems in naming were reported at baseline in more than a third of R-ATL patients and impaired person recognition in more than a third of L-ATL cases. Neuropsychological assessment indicated an even greater percentage of patients showing impairment. Group differences at baseline were attenuated on follow-up. These findings are consistent with evidence that semantic impairments in left- and right-predominant semantic dementia extend, respectively, beyond the verbal and non-verbal domains [7, 21, 26–28].

Behavioural changes too showed overlap in R-ATL and L-ATL groups. All patients showed altered behaviour and the frequency of report for most behaviours did not differ significantly in the two groups. This included disinhibition, loss of empathy, repetitive behaviours, routine or time-bound behaviour, and dietary change. Perseverative, hyper-focussed preoccupations have been consistently highlighted as a feature of R-ATL and have been characterised as altered prioritisation or hedonic valuation [3, 29]. Such behaviour was common in R-ATL but also present in L-ATL. Notably, L-ATL patients frequently showed preoccupation with routine or time-bound behaviour.

The findings reinforce evidence that behavioural change is an integral component of ATL predominant presentations of FTD [8, 9] and that left–right differences in behaviour diminish over the disease course [5]. Behavioural changes, though markers of R-ATL atrophy, are limited in specificity.

An intriguing finding was the greater tendency of R-ATL patients to exhibit an exuberant, and over-demonstrative manner. They enthused, for example, over clinic personnel, the testing environment, and the neuropsychological test stimuli. Their behaviour was reminiscent of the wonderment of young children experiencing a novel event for the first time, and was interpreted, at least in part, as a product of patients’ semantic loss, with environmental stimuli being experienced as novel. Other patients showed blunting of affect and loss of sociability, in keeping with reports that R-ATL patients are emotionally distant [2, 29]. Individual differences highlight behavioural variation within as well as across patient groups.

The overlap in cognitive and behavioural symptoms in R-ATL and L-ATL patients is unsurprising. Despite asymmetries, the temporal lobe atrophy in all but one patient was bilateral. This is consistent with the other reports, which have also shown that left–right asymmetries reduce over the disease course [5, 30, 31]. In the present series, 5 (9%) of 56 patients with anterior temporal lobe degeneration had a symmetric distribution of atrophy at baseline so were excluded from comparative analyses.

### Clinical diagnostic classification

Patients had been diagnosed at the initial assessment based on their predominant clinical phenotype. Both R-ATL and L-ATL groups showed clinical heterogeneity. SD was the most common designation in both groups. The classification was based on the presence of a semantic disorder as the dominant feature. Accompanying behavioural changes characteristic of SD [9] were accepted as part of that SD syndrome. The designation FTD/SD was made when the behavioural changes were the dominant symptom reported at clinical interview, whereas striking semantic loss was demonstrated on neuropsychological assessment, so that neither semantic nor behavioural features predominated overall. This diagnostic classification was more common, albeit not exclusive, to R-ATL cases. A minority of patients in both R-ATL and L-ATL groups were diagnosed with bvFTD or FTD-ALS.

### Diagnostic classification of R-ATL atrophy: two sides of the debate

It has been argued that R-ATL should be considered a separate syndromic variant of FTD [1, 2, 15]. The proposed designation ‘semantic behavioural variant FTD’ [2] captures the combination of behavioural and semantic symptoms. In support of the separation, R-ATL is claimed less likely to be genetically sporadic than its left-sided counterpart, *MAPT, GRN,* and *TARDBP* mutations having been found in a minority of cases [32]. R-ATL has been reported to be associated with a wider range of FTLD pathologies: tau-MAPT and TDP types A and B in addition to the typical TDP type C associated with SD/svPPA [33].

A counterargument can be made, also on clinical, radiological, genetic, and pathological grounds. The substantial cognitive and behavioural overlap in symptoms between R-ATL and L-ATL cases, even at presentation, limits diagnostic specificity. This is as would be expected. Early descriptions of SD, although most often associated with greater left-sided atrophy, recognised the multimodal nature of the conceptual disorder: it progressively affects the understanding of words, non-verbal environmental sounds, faces, objects, smells, tastes, and tactile stimuli [34–38]. Moreover, it involves both hemispheres and hemispheric asymmetries lessen with time [5, 30, 31]. Indeed, it has been argued that the bilateral nature of the disorder is a necessary condition for significant semantic impairment [39, 40]. Some patients in the present series showed symmetrical temporal lobe atrophy at presentation, so fit neither the L-ATL nor R-ATL category. The (relatively) greater behavioural change in R-ATL patients parallels the greater behavioural change reported in bvFTD patients with right compared to left frontal atrophy [41]. The latter difference highlights phenotypic variation as a function of anatomy without implicating a separate diagnostic entity.

The extent of genetic and pathological heterogeneity depends upon the criterion by which patients are defined: clinical or radiological. There is not a one-to-one correspondence between the two. Classification based on clinical phenotype, e.g., SD/svPPA would, a priori, be expected to yield greater homogeneity than that based on radiological appearances, e.g., R-ATL atrophy. A study by Josephs and colleagues [42] illustrates the point. It found R-ATL atrophy to be associated with two distinct syndromes: a behavioural syndrome consistent with bvFTD and mainly associated with *MAPT* tau pathology, and a semantic syndrome consistent with SD and associated with TDP-type-C pathology. Had patients been defined clinically by the syndrome of SD rather than predominant right-temporal atrophy then the pathological substrate would have been uniform. *MAPT* mutations are known to be associated with greater temporal than frontal lobe atrophy [42–45], which is occasionally asymmetric [43]. An association has been reported between FTD-ALS and severe temporal lobe atrophy [46], which, as in this study, may be right or left predominant, the expected pathology being TDP type B [47]. It is notable that in the present study, several L-ATL cases presented with FTD-ALS, which would be strongly predictive of TDP type B rather than type C pathology.

The foregoing suggests that studies that compare SD/svPPA with R-ATL atrophy are not comparing like with like. It also suggests that a classification based on radiological findings risks cutting across distinct genotypes and pathological phenotypes, so has limited diagnostic predictive value. In the present study, L-ATL atrophy was associated with as diverse a range of clinical phenotypes as was R-ATL atrophy, including FTD-ALS. It would be reasonable to suppose that a larger-scale study of patients with L-ATL atrophy, as opposed to SD/svPPA, would elicit varying clinical phenotypes, genetics, and pathology, just as does R-ATL atrophy.

### The influence of terminology

The perceived need for a separate diagnostic category for R-ATL temporal atrophy is undoubtedly influenced by contemporary terminology. The terms SD and svPPA are commonly used interchangeably, yet they have different connotations. svPPA, construed as a form of aphasia [13], cannot encompass variants in which ‘aphasia’ is not the dominant symptom. Hence, a novel designation is required for right-anterior temporal lobe degeneration.

SD, by contrast, construed as a disorder of semantic memory, readily encompasses both left- and right-predominant variants: both involve a progressive breakdown in conceptual knowledge of the world, the varying patterns of breakdown according to anatomy informing understanding of the contributions of the two hemispheres to semantic memory representation. Nomenclature such as “left and right dominant” or “left and right lateralised” SD [5, 24, 28, 48–52] reflects this conception of a distributed disorder of semantic memory, in which changes in behaviour and social functioning represent an integral part. SD also readily accommodates symmetrical presentations of temporal lobe atrophy, whereas the svPPA/R-ATL division does not.

The term svPPA represents an additional clinical conundrum. In the present study, 84% of L-ATL patients met specific diagnostic criteria for svPPA, but only 54% met criteria for all-cause PPA, despite svPPA being a subcategory of PPA. The anomaly arose, because “aphasia”, a pre-requisite for a generic classification of PPA [14], was not invariably the presenting symptom. A parallel anomaly has been reported with respect to other forms of PPA. For example, apraxia of speech, as presenting symptom, is sufficient for a sub-classification of non-fluent variant PPA, but not all-cause criteria for PPA, as speech apraxia constitutes a motor speech disorder rather than aphasia [53].

### Limitations

The study has limitations. It was carried out by retrospective case note review, so that rating of behavioural features and neuropsychological data was dependent on the information obtained at the time of patients’ clinical evaluation. The Visual Object Memory test did not include a verbal equivalent for comparison, complicating interpretation of the greater memory impairment found in R-ATL patients. Thus, it is unclear whether it reflects a primary problem in visual memory or is secondary to impairment in visual object semantics, as suggested above. The absence of group difference in famous face identification, despite more frequent reports by relatives of face identification problems in R-ATL, likely reflects the brief, and hence poorly sensitive, nature (10 items) of the screening test. In a more extensive study, we found significantly greater problems in face identification in patients with R-ATL [21]. Notwithstanding such limitations, a positive feature of the retrospective study is that patients were seen in a single specialist centre and underwent a structured cognitive–behavioural history and neuropsychological assessment protocol independent of imaging. Visually rated atrophy scores are less precise than voxel-based morphometry. However, the use of a well-validated visual rating scale facilitated inclusion of clinical scans carried out across multiple sites, using different image acquisition protocols and is also consistent with the other published reports of R-ATL predominant FTD [4]. In this study, genetic and pathological data were limited, precluding strong conclusions. Nevertheless, the findings are consistent with the notion that clinical phenotype, more than radiological findings, influences genotype and pathology.

## Conclusions

Classification of the frontotemporal dementias remains controversial. In-depth investigation of R-ATL atrophy is valuable in improving understanding of the contribution of the right temporal lobe to cognition and behaviour and in aiding clinical recognition. It does not necessarily follow, however, that it should be regarded as a diagnostic entity distinct from an L-ATL variant. Rather, we propose that a common diagnostic framework for temporal lobe predominant FTD, which takes into account characteristic behavioural changes and person recognition impairment, and acknowledges that aphasia or language impairment per se is not necessarily the presenting feature, would allow for more precise identification of the long-recognised disorder of semantic knowledge, typically genetically sporadic and strongly associated with TDP-type-C pathology. Patient classification needs to be based on clinical phenotype and not radiological findings. Precision in clinical phenotyping informs clinical management and prognosis and predicts underlying pathology.

## Data Availability

The datasets used during the current study are available from the corresponding author on reasonable request.
